# Letter from the Editor in Chief

**DOI:** 10.19102/icrm.2021.121005

**Published:** 2021-10-15

**Authors:** Moussa Mansour


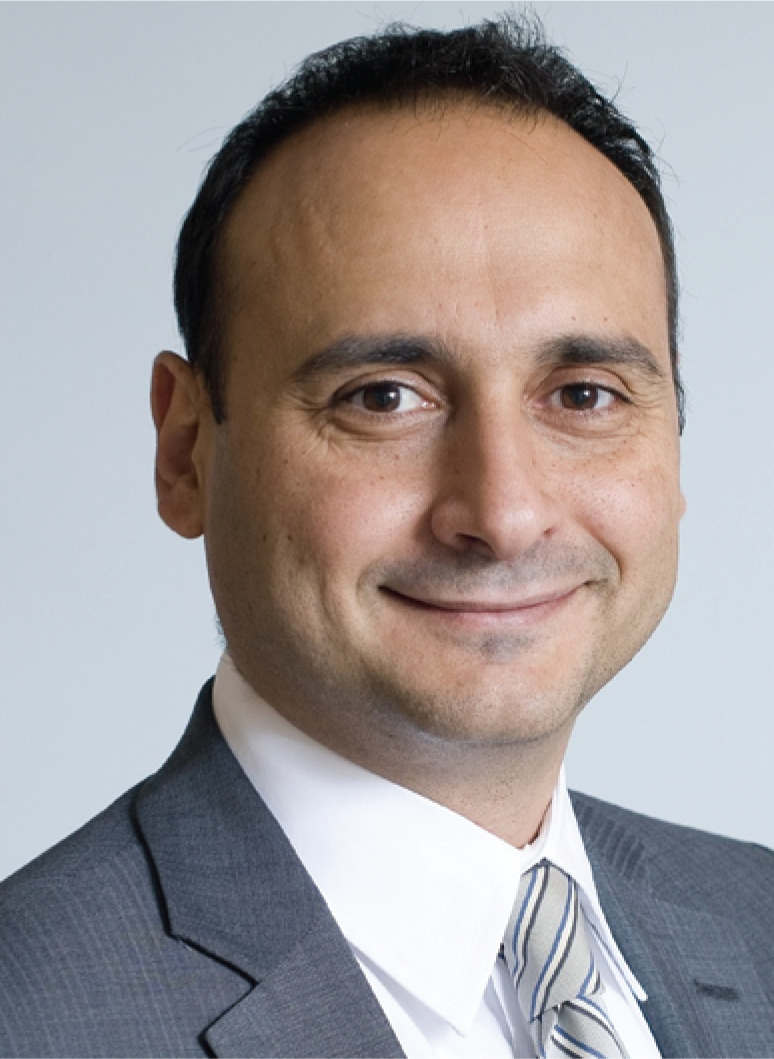


Dear Readers,

This issue of *The Journal of Innovations in Cardiac Rhythm Management* contains an interesting article titled “Epicardial Termination of Left Atrial Appendage Atrial Tachycardia,” in which Polselli et al. describe the ablation of an atrial tachycardia originating from the left atrial appendage (LAA) using an epicardial approach. This article highlights an important topic, which is the role of the LAA in the genesis of atrial arrhythmias.

The LAA has been suspected since at least 2010 to be a trigger or driver for atrial fibrillation (AF).^1^ In addition, the Effect of Empirical LAA Isolation on Long-term Procedure Outcome in Patients With Persistent or Longstanding Persistent AF Undergoing Catheter Ablation (BELIEF) trial,^2^ published in 2016, demonstrated an incremental benefit of isolating the LAA over conventional ablation alone. However, isolation of the LAA has still not become a mainstream adjunct to pulmonary vein isolation (PVI) during AF ablation procedures. One possible reason for the lack of widespread adoption of LAA isolation is the difficulty in achieving this task. The atrial wall of the LAA is thick and typically requires longer ablation lesions at higher power, more than what is necessary to isolate the pulmonary veins. Moreover, pacing is often required to delineate the location of the left phrenic nerve to avoid its injury. Acute recurrence is also often seen during the procedure, necessitating more ablation in order to achieve durable isolation. Collectively, these aspects can add significant time and challenges to the procedure.

Two ongoing randomized clinical trials are expected to shed light on this topic. First, the LAA Ligation Adjunctive to PVI for Persistent or Longstanding Persistent AF (aMAZE) trial (ClinicalTrials.gov identifier no. NCT02513797), which completed enrollment and will be presented at American Heart Association Scientific meeting in November 2021, will help us understand what population(s) might benefit most from LAA isolation and shed light on the safety of this procedure. Another study, the Posterior Wall and LAA Empiric Electrical Isolation for Nonparoxysmal AF (PLEA) (ClinicalTrials.gov identifier no. NCT04216667) trial, is still enrolling patients and aims to show the benefit of radiofrequency isolation of the LAA. If these trials confirm the role of the LAA in AF maintenance, technological advancements, including the use of pulsed ablation, have the ability to make the task easier.

Best regards and I hope that you enjoy reading this issue of *The Journal of Innovations in Cardiac Rhythm Management*.

Sincerely,


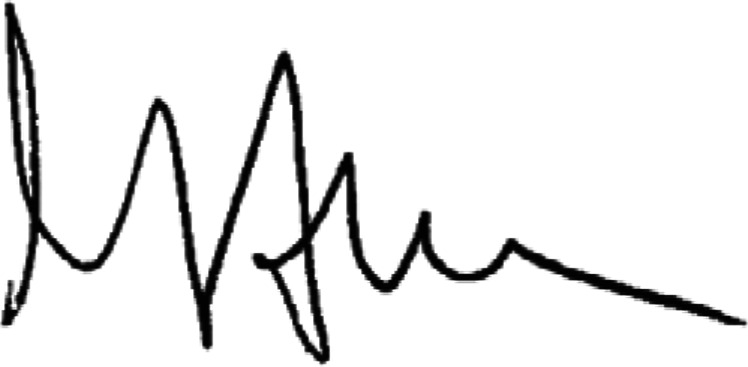


Moussa Mansour, md, fhrs, facc

Editor in Chief

*The Journal of Innovations in Cardiac Rhythm Management*

MMansour@InnovationsInCRM.com

Director, Atrial Fibrillation Program

Jeremy Ruskin and Dan Starks Endowed Chair in Cardiology

Massachusetts General Hospital

Boston, MA 02114
